# Health Care Professional and Caregiver Attitudes Toward and Usage of Medical Podcasting: Questionnaire Study

**DOI:** 10.2196/29857

**Published:** 2022-02-01

**Authors:** Clement Lee, Melissa S Zhou, Evelyn R Wang, Matthew Huber, Katie K Lockwood, Joanna Parga

**Affiliations:** 1 Children's Hospital of Philadelphia Philadelphia, PA United States; 2 University of Pennsylvania Philadelphia, PA United States

**Keywords:** podcasts, social media, caregiver, parent, parenting, education, pediatrics, podcasting, patient education

## Abstract

**Background:**

Podcasts are used increasingly in medicine. There is growing research into the role of podcasts in medical education, but the use of podcasting as a tool for pediatric parent/caregiver health education is largely unexplored. As parents/caregivers seek medical information online, an understanding of parental preferences is needed.

**Objective:**

We sought to explore health care professional and parent/caregiver awareness and views on podcasting as a health education tool.

**Methods:**

This survey study was conducted and distributed via in-person collection from parents/caregivers (≥18 years old) in the waiting room of an academic pediatric primary care clinic, targeted social media promotion, and professional listservs for health care professionals in pediatrics. Statistical analysis included chi-square tests of independence between categorical variables.

**Results:**

In total, 125 health care professionals and 126 caregivers completed the survey. Of those surveyed, 81% (101/125) of health care professionals and 55% (69/126) of parents/caregivers listened to podcasts (*P*<.001). Health care professionals and parents/caregivers listed the same top 3 quality indicators for medical podcasts. Podcast listeners were more likely to have higher incomes and use professional websites for information. The survey elicited a variety of reasons for podcast nonengagement.

**Conclusions:**

Health care professionals appear to be more engaged in medical education podcasts than parents/caregivers. However, similar factors were valued when evaluating the quality of a pediatric podcast: accuracy, transparency, and credibility. Professional websites may be one avenue to increase podcast uptake. More needs to be done to explore the use of podcasts and digital media for medical information.

## Introduction

Since 2006, podcasts have been growing in popularity and influence [1]. Within the medical profession, podcasting is also on the rise. An increasing number of research studies have been conducted on designing podcast content, ensuring the quality of that content, and using audio learning in continuing medical education [2-7]. Although the fields of emergency medicine and critical care appear to have the highest engagement in this medium, there are a number of notable pediatric podcasts that have a growing listenership (PediaCast, Primary Care Perspectives, Peds RAP, Pediatrics On Call) [8-11].

Podcasting as a medium for delivery of medical and health information has many advantages for both parents and health care professionals. Many pediatric podcasts are produced by professionals who elevate evidence-based messaging during a time where antiscience messaging is a widespread problem. Podcasts provide health information in a medium that is easily accessed in times of need, such as overnight when health care providers are not immediately available and parents are looking for digital health information. This is facilitated by podcast archives, which can serve as educational repositories that can be accessed over time [3]. Podcasts also afford real-time and up-to-date learning, so health care professionals can efficiently stay abreast of recent guidelines and field advancements [3,12].

Ongoing assessments of podcasts as a tool for medical education have highlighted their growing popularity—likely due in part to their accessibility and free content—and support continued promotion and content creation in this arena [3,8,13]. However, a review of podcasting and medical education from 2017 showed that no papers were published on the impact of podcasting on patients [2]. This is likely because much of the focus in the literature revolves around continuing medical education for physicians and student learners, and not on parental/caregiver engagement or experience with this medium [14].

In a world that is increasingly digitally connected, our goal was to survey both pediatric health care professionals and parents/caregivers on their views of podcasting as a form of medical education, to explore which podcasts pediatric health care professionals are engaging with for their own education and may be recommending to patients, and to identify how podcasts compare to other digital media in meeting the educational needs of pediatric health care professionals and parents/caregivers.

## Methods

A cross-sectional prospective survey study to collect data on podcasting as a tool for medical education for both health care professionals and parents/caregivers was performed. Survey design and analysis were modeled after the CHERRIES (Checklist for Reporting Results of Internet E-Surveys) format [15]. The target population included both pediatricians at Children’s Hospital of Philadelphia (CHOP) and parents/caregivers bringing their children to a CHOP outpatient pediatrics practice in South Philadelphia (CHOP Primary Care, South Philadelphia). The survey was created in REDCap and survey completion was through the REDCap website for all participants. The survey was disseminated to physicians through an American Academy of Pediatrics and internal CHOP listserv. The study was reviewed by a CHOP Institutional Review Board and granted an exemption due to the anonymous collection of data.

The survey was created and developed by the investigators to answer how podcasting is used for pediatric medical education for health care professionals and parents/caregivers. Based on existing consensus quality indicators, we designed our questions around the 3 themes of credibility (eg, “The information presented by the podcast is accurate”), content (eg, “The content is relevant particularly for my practice/patients”), and design (eg, “The content is conversational/entertaining”) [16]. The complete survey is provided in Multimedia Appendix 1.

The survey was distributed as an open survey without password protection, as no identifying data points were collected on participants. Contact with pediatric health care professionals was made online and the survey was distributed over listservs. Contact with parents/caregivers was made in person by visiting the CHOP Primary Care, South Philadelphia practice. For the recruitment of both health care professionals and parents/caregivers, notice of the survey was also posted on Facebook and Instagram via accounts held by the authors of the study. No incentives were offered for study completion except the ability to add to current scientific literature. The survey was distributed from May 2019 through August 2019. The entire electronic survey consisted of 4 screens without the ability to review entries once they were made. All items on the survey had to be completed for the survey to be submitted.

Descriptive statistics, such as means, medians, and counts/percentages were used to describe the population. Differences between subgroups of the populations (health care providers versus parents/caregivers and podcast listeners versus nonlisteners) were examined using chi-square tests of independence to test for association between categorical variables. A *P* value of >.05 was considered significant. All statistical analyses were done using R (version 3.6.1; R Foundation for Statistical Computing).

## Results

The survey was completed by 251 participants (Table 1). Of the survey respondents, 125 were health care professionals and 126 were parents/caregivers. The median ages of the respondents were 30-39 years for health care professionals, and 30-39 years for parents/caregivers. Notably, parents/caregivers were significantly more likely to be under the age of 30 (*P*=.02). There were no significant differences in gender and self-identified race between the two groups. Health care respondents were more likely to have higher educational attainment and incomes (*P*<.001 for both), with the median annual income being ≥US $200,000 for health care professionals and US $125,000 for parents/caregivers. Of the health care professionals, pediatricians made up the largest group of respondents (95/125, 76%). No health care professionals surveyed used Facebook, Instagram, or YouTube for learning, whereas a larger contingency of parents/caregivers used Facebook (1/126, 1%), Instagram (10/126, 8%), or YouTube (9/126, 7%) for medical information. In addition, 2.4% (3/125) of health care professionals and 0.8% (1/126) of parents/caregivers used Twitter as a source of medical information.

**Table 1:** Demographics.

Characteristic		Podcast listeners (N=170), n (%)		Podcast nonlisteners (N=81), n (%)
**Age cohort (years)**				
	18-20	3 (1.8)	2 (2.5)	
	21-29	26 (15.3)	12 (14.8)	
	30-39	90 (52.9)	33 (40.7)	
	40-49	33 (19.4)	22 (27.2)	
	50-59	15 (8.8)	8 (9.9)	
	≥60	3 (1.8)	4 (4.9)	
Male sex		28 (16.5)		18 (22.2)
**Race**				
	White	144 (84.7)	57 (70.4)	
	Black	6 (3.5)	8 (9.9)	
	Asian	17 (10)	12 (14.8)	
	Other	3 (1.8)	4 (4.9)	
Hispanic or Latino		13 (7.7)		8 (9.9)
**Annual income (US $)**				
	0-9999	2 (1.2)	6 (7.4)	
	10,000-24,999	3 (1.8)	4 (4.9)	
	25,000-49,999	4 (2.4)	6 (7.4)	
	50,000-74,999	11 (6.5)	13 (16.1)	
	75,000-99,999	9 (5.3)	4 (4.9)	
	100,000-124,999	18 (10.6)	5 (6.2)	
	125,000-149,999	14 (8.2)	3 (3.7)	
	150,000-174,999	15 (8.8)	5 (6.2)	
	175,000-199,999	17 (10)	6 (7.4)	
	≥200,000	68 (40)	19 (23.5)	
	Decline to answer	9 (5.3)	10 (12.4)	
**Marital status**				
	Single	25 (14.7)	20 (24.7)	
	Married	141 (82.9)	56 (69.1)	
	Widowed	0 (0)	0 (0)	
	Divorced	3 (1.8)	1 (1.2)	
	Separated	0 (0)	4 (4.9)	
	Other	1 (0.6)	0 (0)	
**Education**				
	Less than high school	1 (0.6)	3 (3.7)	
	High school degree/equivalent	4 (2.4)	10 (12.4)	
	Some college	6 (3.5)	8 (9.9)	
	Associate	2 (1.2)	9 (11.1)	
	Bachelor	25 (14.7)	8 (9.9)	
	Graduate	132 (77.7)	43 (53.1)	
**Role**				
	Health care provider	101 (59.4)	24 (29.6)	
	Parent/caregiver	69 (40.6)	57 (70.4)	

Although over half of respondents in both groups listened to podcasts, far more health care professionals engaged in the medium (101/125, 81%) than parents/caregivers (69/126, 55%; *P*<.001). Of the parents/caregivers who listened to podcasts, more than half (58/69, 84%) had a bachelor's degree or higher and most of the respondents were White (47/69, 68%). Approximately 5% (6/126) of parents/caregivers had never heard of a podcast. As a whole, podcast listeners were more likely to have higher incomes (*P*=.001). Those who used podcasts were more likely to use professional websites as additional sources of information, whereas those who did not favored YouTube (*P*<.001). Both health care professionals and parents/caregivers agreed on the top 3 desired qualities of a podcast: accuracy of the information presented, a distinction made on the podcast between fact and opinion, and podcast host qualifications (Textbox 1).

**Podcast qualities valued by listeners, ranked by percentage of respondent 5/5 (“always”) ratings.**

**Physicians**

1. Accuracy of information (107/125, 85.6%)
2. Authors are qualified (98/125, 78.4%)
3. Fact vs opinion is clear (86/125, 68.8%)
4. Professionalism (65/125, 52%)
5. Relevancy to patients (64/125, 51.2%)

**Caregivers**

1. Accuracy of information (77/126, 61.1%)
2. Authors are qualified (69/126, 54.8%)
3. Fact vs opinion is clear (65/126, 51.6%)
4. Content is entertaining (47/126, 37.3%)
5. Professionalism/relevancy (39/126, 31%)

The most commonly listed reason for not listening to podcasts was a lack of time (19/125, 15.2% of providers and 36/126, 28.6% of parents/caregivers); however, a majority of respondents who did not listen to podcasts cited other reasons, including finding them too slow, being overwhelmed by the options, lacking a routine for them, and not finding them entertaining. Even though most health care professionals listened to podcasts themselves, only 21/125 (17%) recommended podcasts to their patients as a form of education.

## Discussion

In a survey completed by 251 health care professionals and patients’ parents/caregivers, there were significant differences in the pattern of podcast use between the two groups. Although a majority of each group surveyed listened to podcasts, health care professionals were significantly more likely to use podcasts as an educational medium. Given the extensive amount of research and quality improvement that has gone into the medical podcasting sphere, this is perhaps not a surprising finding [4,16].

Both educational status and income level were higher in those who listened to podcasts. Lower income in the parent/caregiver group compared to the health care professional group resulting in fewer resources may explain the disparity seen in podcast use between the two groups of individuals. Smartphones have been shown to be the most popular device for listening to podcasts: 65% of podcast consumers listen to podcasts on mobile devices, compared to 25% on computers or laptops, and 10% on smart speakers [1]. In low-income households with incomes below US $30,000 a year, 29% do not own a cell phone and 44% do not have broadband internet services, and therefore have limited access to podcasts [17].

Podcast listeners, whether health care providers or parents/caregivers, tended to agree on the 3 top qualities they look for in medical podcasts, identified in this study as the following: accuracy of the information presented, a distinction made on the podcast between fact and opinion, and podcast host qualifications. This was a surprising finding, given previous literature suggesting health care educators seemed to require less stringent qualifications for podcasts as compared to other media, with coherence, citations, and expertise required for blogs but not for podcasts [4]. Accessibility across multiple platforms, identified as a major criterion for a quality podcast in that study, was not one of the top 3 qualities identified in our study. We hypothesize this could relate to advancements in smartphone and streaming technology in the past few years making podcasts more accessible overall.

Our study shows a high rate of listenership among surveyed health care providers (101/125, 81%), which is consistent with numbers from other recent studies showing podcast listenership rates among internal medicine and emergency medicine residents of 59% and 89%, respectively [18,19]. Medical podcasts represent an accessible and flexible means of continuing medical education (CME) for health care professionals who have completed formal training, as well as for trainees who seek supplementation to more traditional avenues of medical education. Many currently available pediatric podcasts offer CME credits for their audience, such as Peds RAP [20], and cover seasonal and salient topics of interest, such as diagnosing and treating “long COVID” in children (Pediatrics on Call Episode 64 [21]). There are also examples of national societies as well as residents/fellows creating podcasts with specialty-specific content (eg, “Bowel Sounds: The Pediatric GI Podcast” [22]), showcasing the ability of the medium to serve diverse medical education needs [23,24]. For trainees who attend required didactics as live lectures or case discussions, often 30-60 minutes in length and during the workday, a podcast covering the same material that can be archived and listened to on a flexible timetable is an invaluable self-directed learning resource that gives listeners the discretion to engage when they so desire. Further studies on the efficacy of podcasting as a medical education tool should be explored.

Interestingly, in our study, while a large majority of health care professionals listen to educational podcasts, only 17% (21/125) of them recommend podcasts as a resource to patients or parents/caregivers. Although this study did not specifically address the reasons why they did not refer parents/caregivers to these podcasts, this may be due to the types of podcasts being consumed and the motivations behind their use. An iTunes search of pediatric podcasts at the time of writing uncovered 25 podcasts, only 5 of which included parents/caregivers in their targeted audience. Instead, the majority of podcasts aim to be “edutainment,” or medically educational entertainment, for physicians [24]. Pediatricians in remote hospitals have previously reported that podcasts help them stay connected to colleagues in their field and up to date with recent practice patterns [25]. More recently, a subset of medical podcasts has targeted early-stage trainees with the goal of increasing exposure to different areas of interest within pediatrics (eg, Charting Pediatrics [26]). The style and content of these pediatric podcasts cater more to health care professionals and trainees than to patients and parents/caregivers, which likely factors into why so few providers surveyed indicated they would recommend medical podcasts to patients and their families. Health care professionals may not want to direct their patients toward resources that use medical jargon or present health care information that is difficult for a family to interpret in the context of their individual situations. Health care professionals value the physician-patient relationship and may wish to preserve direct communication of medical advice between the provider and patient/guardian. Finally, the electronic medical record has been designed to facilitate the inclusion of more traditional paper printouts for patient education, a more historic form of guidance and a tested resource. Given the time-pressured environment of outpatient medicine and the lack of a centralized medical podcast repository, describing and facilitating access to podcasts would place an additional cognitive load on the health care professional, making it less likely that such an action would be taken. Further qualitative research may elucidate the reasoning behind provider hesitancy to recommend podcasts to patient families. This information would guide podcast creators on how to broaden their audience to include patients and parent/caregivers in addition to health care professionals.

The vast majority of health care professionals and parents/caregivers did not use social media platforms as a source of medical information, despite their known popularity in the personal lives of both groups. Of note, the listed social media platforms (Facebook, Instagram, Twitter, YouTube) lack the quality indicators our study’s participants cited as most important when they seek educational podcasts, such as accuracy, transparency, and a qualified host. This is an interesting contrast to prior surveys that have identified a high percentage of health professionals who use social media for education [27]. Differences may be due to the demographic surveyed (eg, attending physicians versus graduate medical trainees) or survey wording that precluded the consideration of subconscious consumption of information on social media.

Our study is subject to the many limitations inherent to an online survey. There was no way to ensure the survey was only filled out once per respondent. Recruitment through social media may make the data regarding other sources of information difficult to interpret (eg, a patient responding to the survey through Facebook evidently uses Facebook for information). The survey was internally created and not a previously validated survey. A popular pediatric podcast is hosted by one of the authors, so an internally distributed survey may skew the physician population toward higher listenership. In addition, because the survey was distributed in one clinic within a hospital network in one city, the results have narrow generalizability, although the demographics of the listeners surveyed were typical of national podcast listeners [1]. Of note, the sampled parents/caregivers were mostly White, though the clinic where they were surveyed serves a primarily non-White demographic (26% White). This highlights potential biases in the administration of the survey, such as selection bias, language barriers (the survey was only offered in English), nonresponse bias, or the use of a White surveyor. Next steps may attempt to address this by reaching a more representative sample of the communities served. Other future work might include examining the motivations of physician versus parent/caregiver in listening to podcasts, qualitative studies on effective presentation of podcast information to parent/caregivers to promote message uptake, and optimizing dissemination of podcasts as an education tool at different points of contact with the health care system (primary care versus the emergency room versus the hospital setting).

In summary, although many health care professionals and parents/caregivers alike use podcasts as a source of information, there is unrealized potential for more engagement with this medium that health care professionals can help to facilitate. With the evolving world of data overload, targeted efforts at improving use of podcasts in patients and parents/caregivers may offer this population a source of updated, accurate medical information. Furthermore, awareness of trustworthy messaging is urgently needed in the setting of rising antivaccine and antiscience sentiment. There are data supporting the sustainability of podcasts over time, suggesting that a medical professional’s recommendation of podcast use, if accepted, may lead to lasting use and ongoing education for parents/caregivers [28,29]. This may be particularly salient during the COVID-19 pandemic and the increasing use of telemedicine and other virtual ways of delivering health care and medical information, particularly in remote areas, where reliable medical information is not as easy or as convenient to access. Given that both health care providers and parents/caregivers primarily used professional websites to obtain medical information, podcasters should consider partnering with professional websites among other creative solutions to increase uptake of the medium. Educational podcasts that can offer accurate, transparent, and credible medical information to health care providers, patients, and their families are likely to continue to grow as an enduring form of medical education.

## Appendix

Multimedia Appendix 1 Sample of survey administered to participants.

## Abbreviations

CHERRIES: Checklist for Reporting Results of Internet E-Surveys
CHOP: Children’s Hospital of Philadelphia
CME: continuing medical education
