# Supplementary material for: Health Care Professional and Caregiver Attitudes Toward and Usage of Medical Podcasting: Questionnaire Study
Source: JMIR Pediatr Parent. 2022 Feb 1;5(1):e29857. doi: 10.2196/29857 (PMC8848225; doi:10.2196/29857)
Supplement: Multimedia Appendix 1 [file pediatrics_v5i1e29857_app1.docx]

**Supplementary Appendix**

**Survey for Providers**

1. What is your provider role?
   1. Pediatrician
   2. Physician assistant
   3. Medical assistant
   4. Nurse practitioner
   5. Nurse
   6. Other:
2. What category includes your age?
   1. 18-20
   2. 21-29
   3. 30-39
   4. 40-49
   5. 50-59
   6. 60 or older
3. What race do you identify as?
   1. White
   2. Black or African American
   3. American Indian or Alaskan Native
   4. Asian
   5. Native Hawaiian or other Pacific islander
   6. From multiple races
   7. Other:
4. Are you Hispanic?
   1. Yes
   2. No
5. What gender do you identify as?
   1. Male
   2. Female
   3. Other:
6. What is your marital status?
   1. Married
   2. Widowed
   3. Divorced
   4. Separated
   5. Never married
7. What is your highest level of education?
   1. Less than high school degree
   2. High school degree or equivalent (e.g., GED)
   3. Some college but no degree
   4. Associate degree
   5. Bachelor’s degree
   6. Graduate degree
8. How much total combined money did all members of your household earn last year?
   1. $0 to $9,999
   2. $10,000 to $24,999
   3. $25,000 to $49,999
   4. $50,000 to $74,999
   5. $75,000 to $99,999
   6. $100,000 to $124,999
   7. $125,000 to $149,999
   8. $150,000 to $174,999
   9. $175,000 to $199,999
   10. $200,000 and up
   11. Prefer not to answer
9. Do you listen to podcasts?
   1. If no
      1. Why don’t you listen to podcasts?
         1. I’ve never heard of a podcast
         2. I don’t know where to access podcasts
         3. I don’t like listening to the radio/audio content
         4. I don’t have time to listen
         5. Other:
   2. If yes
      1. Do you listen to any pediatric podcasts in order to learn more about a subject?
         1. If yes: which ones?

(iii). Why do you listen to pediatric podcasts?

1. I learn best from audio mediums
2. I like the flexibility of this medium over lectures, webinars, conferences, or reading
3. I am an avid listener of podcasts on a variety of topics
   - 1. Do you ever recommend pediatric podcasts to patients to help educate them on a subject?
        1. If yes: which ones?
     2. What do you use to determine the quality of a pediatric podcast (all answers on a Likert scale 1=never, 2=rarely, 3=something, 4=very often, 5=always)?
        1. Conflicts of interest are clearly stated
        2. The identity of the podcast’s author and who created the episode is clearly stated
        3. The authors are well qualified to provide information on this topic
        4. A clear distinction is made between fact and opinion
        5. The information presented by the podcast is accurate
        6. The podcast is easily accessible among various audio platforms
        7. References are clearly cited and the statements on the podcast reflect the references
        8. The content is professional
        9. The content is conversational/entertaining
        10. The content is relevant particularly for my practice/patients
        11. Other:
4. Besides podcasts, what other forms of digital media do you use to get medical information?
   1. Facebook
   2. Instagram
   3. Twitter
   4. YouTube
   5. Websites by professional organizations (ie. healthychildren.org by the American Academy of Pediatrics)
   6. Other (please list the name of any resources you use frequently here):
5. What other forms of digital media do you refer patients to for medical information?
   1. Facebook
   2. Instagram
   3. Twitter
   4. YouTube
   5. Websites by professional organizations (ie. healthychildren.org)
   6. Other (please list the name of any resources you frequently refer patients to here):
6. Thank you for your participation. If you have any further comments on the utilization of podcasts or digital media in medicine, please leave them here:

**Survey for Patients**

1. What category includes your age?
   1. 18-20
   2. 21-29
   3. 30-39
   4. 40-49
   5. 50-59
   6. 60 or older
2. What race do you identify as?
   1. White
   2. Black or African-American
   3. American Indian or Alaskan Native
   4. Asian
   5. Native Hawaiian or other Pacific islander
   6. From multiple races
   7. Other:
3. What gender do you identify as?
   1. Male
   2. Female
   3. Other:
4. What is your marital status?
   1. Married
   2. Widowed
   3. Divorced
   4. Separated
   5. Never married
5. What is your highest level of education?
   1. Less than high school degree
   2. High school degree or equivalent (e.g., GED)
   3. Some college but no degree
   4. Associate degree
   5. Bachelor degree
   6. Graduate degree
6. How much total combined money did all members of your household earn last year?
   1. $0 to $9,999
   2. $10,000 to $24,999
   3. $25,000 to $49,999
   4. $50,000 to $74,999
   5. $75,000 to $99,999
   6. $100,000 to $124,999
   7. $125,000 to $149,999
   8. $150,000 to $174,999
   9. $175,000 to $199,999
   10. $200,000 and up
   11. Prefer not to answer
7. Do you listen to podcasts?
   1. If no
      1. Why don’t you listen to podcasts?
         1. I’ve never heard of a podcast
         2. I don’t know where to access podcasts
         3. I don’t like listening to the radio/audio content
         4. I don’t have time to listen
         5. Other:
   2. If yes
      1. What podcasts do you listen to? (fill in the blank)
      2. Do you listen to any pediatric podcasts in order to learn more about children’s health or well-being?
         1. If yes: which ones?
      3. Do you ever recommend pediatric medical podcasts to family or friends?
         1. If yes: which ones?
      4. What do you use to determine the quality of a pediatric medical podcast (all answers on a Likert scale 1=never, 2=rarely, 3=something, 4=very often, 5=always)
         1. Conflicts of interest are clearly stated
         2. The identity of the podcast’s author and who created the episode is clearly stated
         3. The authors are well qualified to provide information on this topic
         4. A clear distinction is made between fact and opinion
         5. The information presented by the podcast is accurate
         6. The podcast is easily accessible among various audio platforms
         7. References are clearly cited and the statements on the podcast reflect the references
         8. The content is professional
         9. The content is conversational/entertaining
         10. The content is relevant to my child’s health or my health
         11. Other:
8. Besides podcasts, what other forms of digital media do you use to get pediatric medical information?
   1. Facebook
   2. Instagram
   3. Twitter
   4. YouTube
   5. Websites by professional pediatric organizations (ie. healthychildren.org by the American Academy of Pediatrics)
   6. Other (please list the name of any resources you use frequently here):
9. Thank you for your participation. If you have any further comments about how you use podcasts or digital media to learn about medicine, please leave them here:
